# Molecular and metabolic orchestration of the lymphatic vasculature in physiology and pathology

**DOI:** 10.1038/s41467-023-44133-x

**Published:** 2023-12-16

**Authors:** Nieves Montenegro-Navarro, Claudia García-Báez, Melissa García-Caballero

**Affiliations:** 1 Department of Molecular Biology and Biochemistry, Faculty of Sciences, University of Málaga, Andalucía Tech, Málaga, Spain; 2 Instituto de Investigación Biomédica de Málaga y Plataforma en Nanomedicina (IBIMA Plataforma BIONAND), Málaga, Spain

**Keywords:** Lymphangiogenesis, Signal transduction, Cell biology, Translational immunology

## Abstract

Lymphangiogenesis refers to the generation of new lymphatic vessels from pre-existing ones. During development and particular adult states, lymphatic endothelial cells (LEC) undergo reprogramming of their transcriptomic and signaling networks to support the high demands imposed by cell proliferation and migration. Although there has been substantial progress in identifying growth factors and signaling pathways controlling lymphangiogenesis in the last decades, insights into the role of metabolism in lymphatic cell functions are just emerging. Despite numerous similarities between the main metabolic pathways existing in LECs, blood ECs (BEC) and other cell types, accumulating evidence has revealed that LECs acquire a unique metabolic signature during lymphangiogenesis, and their metabolic engine is intertwined with molecular regulatory networks, resulting in a tightly regulated and interconnected process. Considering the implication of lymphatic dysfunction in cancer and lymphedema, alongside other pathologies, recent findings hold promising opportunities to develop novel therapeutic approaches. In this review, we provide an overview of the status of knowledge in the molecular and metabolic network regulating the lymphatic vasculature in health and disease.

## Introduction

The lymphatic system is comprised of an open, unidirectional, and low-pressure network of lymphatic vessels, along with primary and secondary lymphoid organs, such as spleen, thymus and lymph nodes^1–3^. It maintains the interstitial fluid volume and composition for tissue homeostasis, participates in the intestinal absorption of dietary fats and vitamins, and is essential for immunosurveillance and immunomodulation^4^. The formation of the lymphatic network from the preexisting vasculature is a highly controlled process termed lymphangiogenesis^1–3^. Despite being crucially active during embryogenesis, lymphatic growth in adult tissues is limited to particular circumstances and locations, including the female reproductive cycle, wound healing and pathological events like tumors and inflammation^5^. Moreover, impaired lymphatic formation and function also contributes to lymphedema progression^6^. Although most of the studies on lymphatic dysfunction have focused on cancer and lymphedema, lymphatics have been recently associated with neurological and neurodegenerative disorders, inflammatory bowel disease, cardiovascular disease, metabolic syndrome and glaucoma^1^.

All throughout the lymphatic system, lymphatic vessels are lined by lymphatic endothelial cells (LEC), which are endothelial cells (EC) displaying specific transcriptional, molecular and metabolic programs accountable for the lymphatic system’s many and essential functions^4^. The morphology of lymphatic vessels is often uniquely tied with their distinct functions and sizes^4^. The most basic constituent of the lymphatic vasculature are lymphatic capillaries, which are highly permeable structures, formed exclusively by a monolayer of discontinuously linked LECs^6^. Due to their structure, lymphatic capillaries allow for the filtration of interstitial fluid and the formation of lymph. Lymph is then transported to larger vascular structures named pre-collecting and collecting vessels, which are composed of a continuous, airtight monolayer of LECs, covered with specialized smooth muscle cells (SMC)^6^. SMCs not only guarantee the flow of lymph and its unidirectionality, but also participate in the morphogenesis of the lymphatic system^1,7,8^. On the other hand, SMC contractile activity depends on the physical, biochemical, and immunological signals from the environment, such as nitric oxide (NO) synthesized by the endothelium^1,7,8^.

So far, the molecular regulation of lymphangiogenesis has been extensively studied and reviewed, from the transcriptional control of lymphatic fate acquisition in embryonic stages to the growth factors and signaling pathways controlling developmental and adult lymphangiogenesis^6,9–12^. In the last decade, an involvement of metabolism in lymphatic formation and function has been elucidated. In fact, it was first described the crucial implication of fatty acid oxidation (FAO) and glycolysis in lymphatic vascular development^13,14^, and recent studies have revealed how other metabolic pathways, such as fatty acid synthesis^15^, ketone bodies oxidation (KBO)^16^, oxidative phosphorylation (OXPHOS)^17^ and amino acid metabolism^18,19^ regulate lymphatic function as well.

Under pathological conditions, especially in cancer, ECs experience a switch from a quiescent state to a highly proliferative and active state (“(lymph)angiogenic switch”)^20^. This reprogramming is not only driven by alterations in (lymph)angiogenic growth factors, but also by a “metabolic switch”^20^. Since the clinical benefits of inhibiting (lymph)angiogenic growth factor networks for various disorders have not been entirely successful^21^, efforts are needed to identify novel targets. In this context, LECs associated with specific pathological states display an altered metabolism that may be exploited to discover promising therapeutic targets. In this review, we highlight the molecular regulation of the lymphatic system, the mechanisms governing LEC metabolism in health and disease, and the clinical possibilities of exploiting the molecular and metabolic network underlying lymphangiogenesis.

## Molecular drivers of the lymphangiogenic process

Given that lymphangiogenesis is such a relevant process in the maintenance of systemic tissue homeostasis, it is not surprising that lymphatic vessel formation must be tightly regulated. This regulation encompasses not only its establishment and maturation but also the maintenance of lymphatic identity and function in postnatal stages. Albeit less explored than angiogenesis, the molecular network coordinating lymphangiogenesis has been extensively studied over the last decades^6,9–12^. Different molecular players, including transcription factors, growth factors, and cytokines, have been described as key mediators of embryonic and adult lymphangiogenesis. In the following subsections, the main lymphangiogenic molecular drivers are described in more detail.

### SOX18/COUP-TFII/PROX1 axis: the main transcriptional regulator of embryonic lymphangiogenesis

**Fig. 1:**
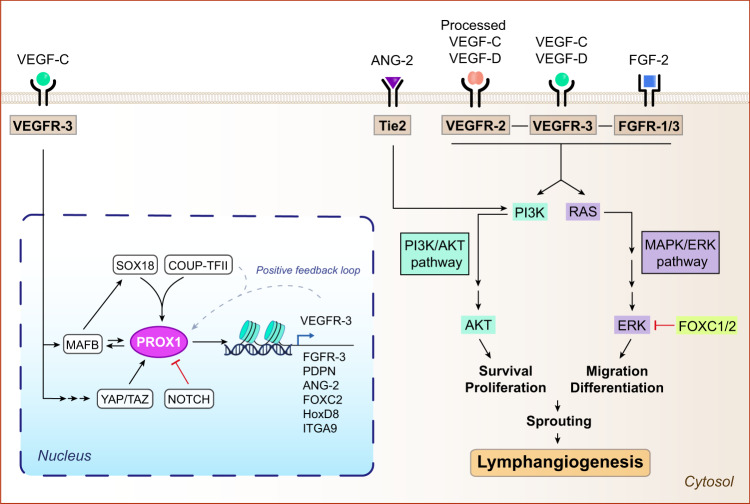
Molecular regulation of lymphatic growth and function. At transcriptional levels, *PROX1* expression is positively regulated by the transcription factors SOX18, COUP-TFII, MAFB, and YAP/TAZ signaling pathway, and negatively regulated by the Notch signaling pathway. In turn, PROX1 promotes the expression of lymphangiogenic genes, including VEGFR-3, which induces *PROX1* expression alongside COUP-TFII through a positive feedback loop. At cytokine/receptor levels, the interaction of VEGF-C/D and FGF-2 with their cognate receptors promote lymphangiogenesis by activating the PI3K/AKT and the MAPK/ERK pathways, consequently stimulating cell survival, proliferation, differentiation, migration, and sprouting. ANG-2 exclusively stimulates the PI3K/AKT pathway. FOXC1/2 negatively regulates lymphangiogenesis by interfering with the MAPK/ERK pathway. Figure was created with Adobe Illustrator and the DNA strand was created with BioRender.com.

PROX1 is the major transcription factor driving the establishment of lymphatic identity and the initiation of embryonic lymphangiogenesis^22^. During the E9.5 stage of mammalian development, *Prox1* expression is detectable in a subset of venous endothelial cells (VEC) located in the cardinal vein^23^. Such *Prox1* expression drives the differentiation of VECs into LECs, which undergo further development to form the lymphatic system^22^. Upstream of *Prox1*, two transcription factors, SOX18 (SRY –Sex Determining Region Y– Box 18) and COUP-TFII (Chicken Ovalbumin Upstream Promoter Transcription Factor II –also known as NR2F2–), are expressed in the anterior cardinal vein during initial lymphatic fate specification and directly promote *Prox1* expression by binding to its promoter^24,25^. More recently, the musculoaponeurotic fibrosarcoma oncogene homolog B (MAFB) transcription factor has also been shown to propagate *Prox1*, *Sox18*, and *Coup-TFII* expression^26,27^ (Fig. 1).

The functions of PROX1 have been extensively studied using both in vitro and in vivo genetic models^28,29^. Consistent with its role as a transcription factor, PROX1 directly regulates the expression of several lymphatic genes by binding to their promoter through a C-terminal DNA-binding domain^30^. The main transcriptional target of PROX1 is *Flt4*, which encodes the vascular endothelial growth factor receptor (VEGFR)−3 (VEGFR-3), a master regulator of lymphangiogenesis^31^. During development, PROX1^+^ and VEGFR-3^+^ LECs migrate from the embryonic vein and proliferate to form a capillary-like structure, organizing themselves into lymphatic sacs and a primitive lymphatic plexus^29^. Nonetheless, additional studies have revealed novel transcriptional targets of PROX1, including the fibroblast growth factor receptor 3 (FGFR-3), podoplanin (PDPN), angiopoietin-2 (ANG-2), forkhead box protein C2 (FOXC2), homeobox D8 (HoxD8) and integrin-α9 (ITGA9)^28,30,32^. Following induction, *Prox1* expression is sustained through a positive feedback loop with VEGFR-3 and COUP-TFII, allowing the maintenance of a constant *Prox1* expression that explains its designation as a constitutive marker of LECs in adult tissues^33^ (Fig. 1). Interestingly, the mechanisms underlying how VEGFR-3 regulates *Prox1* expression have been partly explained with the identification of YAP and TAZ transcription factors, which modulate *Prox1* expression during lymphatic vascular development^34^ (Fig. 1). Therefore, PROX1 is not only crucial during early lymphatic specification, but also for the maintenance of lymphatic identity and lymphangiogenesis in postnatal stages.

### Growth factors and cytokines involved in lymphatic regulation

#### VEGF-C/D/VEGFR-3 axis as a key modulator of lymphangiogenesis

The VEGF-C/D/VEGFR-3 signaling pathway is the major regulator of both physiological and pathological lymphangiogenesis^35^. PROX1/COUP-TFII-mediated induction of VEGFR-3 is a crucial requisite for increasing the susceptibility of LECs to VEGF-C/D and the activation of the VEGFR-3 signaling pathway^36,37^. Resembling the key role of VEGF-A in blood vessel formation, VEGF-C/D serve as crucial secretory regulators of lymphangiogenesis and are produced by diverse cell types, such as tumor, immune and LECs^11,38^. Although VEGF-C/D reach their maximum effects after interacting with VEGFR-3, proteolytically processed VEGF-C/D can also bind and activate VEGFR-2^39,40^. The VEGF-C/D/VEGFR-3 interaction induces the dimerization and activation of the receptor through autophosphorylation of specific tyrosine residues within the VEGFR-3 cytoplasmic domain^11^. The main downstream event following VEGFR-3 activation is the stimulation of the phosphoinositide-3-kinase-protein kinase B/Akt (PI3K-PKB/AKT) and the mitogen-activated protein kinase/ERK (MAPK/ERK) signaling pathways, which further promote LEC survival, proliferation, differentiation and migration (Fig. 1). These processes culminate in the sprouting and growth of new lymphatic vessels. During development, the embryonic lymphatic vessels separate from the original veins for expanding the primary lymphatic plexus and remodeling into lymphatic capillaries and collecting lymphatic vessels^23^. Subsequently, lymphatic maturation and valve formation is regulated by FOXC2 and Notch1 signaling^41^ (Fig. 1). It is worth mentioning that lineage-tracing experiments have shown that LECs may arise from non-venous origin as well^42^. For example, evidence indicates that other cellular lineages contributing to mesenteric, cardiac and dorsal dermal lymphatics are the paraxial mesoderm^43^, the hemogenic endothelium^44^, the second heart field^45^ and dermal capillaries^46^.

The VEGF-C/VEGFR-3 network is an indispensable component during lymphangiogenesis. Nevertheless, additional cytokine/receptor systems, including the fibroblast growth factor (FGF) and angiopoietin (ANG) cytokine families, are engaged in this process in concert with their cognate receptors, as explained below.

#### Beyond VEGF: the role of FGF/FGFR and ANG2/TIE2

The FGF family comprises 22 members. Among which, FGF-2 has been shown to stimulate lymphatic development following its interaction with FGFR-1 and/or FGFR-3^38^, FGFR-1 is considered the predominant FGFR in LECs and *Fgfr-1* knockdown is sufficient to impair LEC proliferation, migration, and tube formation in vitro^47^, and FGF-2 binding to FGFR-3 also promotes in vitro lymphangiogenesis^32,38^. By contrast, FGFR-1 loss during lymphatic growth in vivo can be compensated by FGFR-3, which is upregulated in LECs during lymphatic differentiation^48^. Importantly, Yu et al. revealed that, while genetic deletion of lymphatic *Fgfr1* has no impact, double knockout of both *Fgfr*1 and *Fgfr3* triggers profound lymphatic vascular defects in mice^14^.

Mirroring the downstream effects of the VEGF-C/VEGFR-3 signaling pathway, the activation of the FGF-2/FGFR-1/FGFR-3 axis induces LEC proliferation, migration and survival through the stimulation of PI3K/AKT and MAPK/ERK signaling pathways^38^ (Fig. 1). In fact, FGF-2 implantation in mice corneas was reported to exert a pro-lymphangiogenic effect^49^ that could be reverted with the use of anti-VEGFR-3 antibodies^50^. These results suggested a synergistic relation between VEGFR-3 and FGFR signaling pathways, as shown by Cao and colleagues in murine corneas and tumor microenvironment (TME)^51^.

In contrast with VEGF and FGF, the role of Ang family in lymphangiogenesis has been less explored. Ang family comprises 3 members in humans: ANG1, ANG2 and ANG4, with human ANG4 corresponding to Ang3 in mice^38^. ANG1 and ANG2 mediate their downstream effects by interacting with TIE2 receptor (also known as TEK), and this interaction is important for the maintenance of lymphatic junctional stability^52^. Following ANG2/TIE2 binding, the PI3K/AKT pathway is activated, consequently promoting LEC proliferation and survival^38^ (Fig. 1).

Although we have referred to a single molecular profile of LECs, organ-specific heterogeneity should also be considered. Recent evidence shows inter-tissue differences in the lymphatic and blood vasculature at a functional and molecular level^53,54^. For instance, unlike other LECs, brain mural LECs express a perivascular macrophage marker (mannose receptor 1) and form non-lumenized vessels^55^. Another example of differential gene expression is represented by LECs from the Schlemm’s canal, which lack LYVE1 and PDPN lymphatic marker expression^56^. The transcriptional signature of intestinal LECs also differs from that of their dermal counterparts in vitro^57^.

**Fig. 2:**
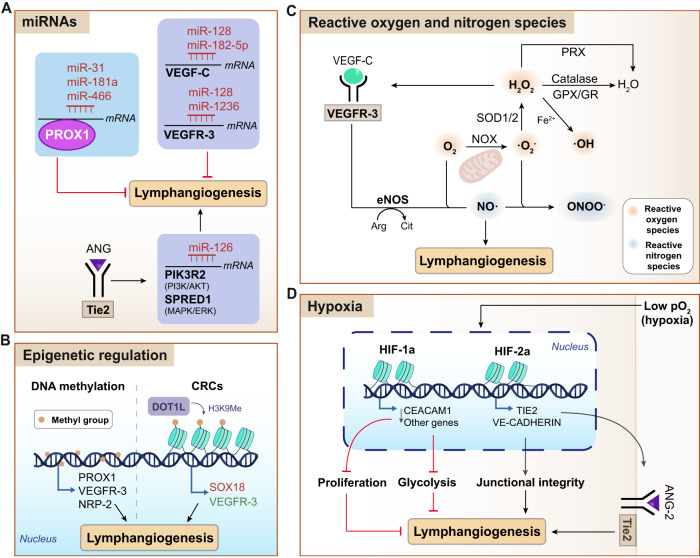
Emergent modulators of lymphatic vessel formation: miRNAs, epigenetics, reactive and nitrogen oxygen species and hypoxia. **A** miRNAs. miR-31, miR-181a and miR-466 can target *PROX1*; miR-128 and miR-182-5p can target *VEGF-C*; and miR-128 miR-1236 can target *VEGFR-3* to negatively regulate lymphangiogenesis. By interacting with Tie2, ANG-2 regulates miR-126 and promotes lymphangiogenesis by targeting genes involved in the PI3K/AKT and MAPK/ERK signaling pathways (PIK3R2 and SPRED1, respectively). **B** Epigenetic regulation. The epigenetic regulation of lymphangiogenesis involves DNA methylation and histone methylation through chromatin remodeling complexes (CRC), namely DOT1L, to promote the expression of lymphatic genes (PROX1, VEGFR-3, NRP-2; and SOX18 and VEGFR-3, respectively). **C** Reactive and nitrogen oxygen species. Lymphatic function is modulated by hydrogen peroxide (H_2_O_2_), which is generated after O_2_ reduction in the mitochondria or by NADPH oxidases (NOX) that generate superoxide anion (·O_2_^-^), and subsequently H_2_O_2_ via superoxide dismutase (SOD1/2). Afterwards, H_2_O_2_ can be reduced to H_2_O via catalase, glutathione peroxidase (GPX)/glutathione reductase (GR) or peroxiredoxin (PRX). H_2_O_2_ can activate VEGFR-3, with the subsequent activation of eNOS and nitric oxide (NO·) production, which ultimately stimulates lymphangiogenesis. **D** Hypoxia. Hypoxia regulates lymphangiogenesis through HIF-1α and HIF-2α induction. HIF-1α reduces lymphangiogenesis via *CEACAM1* (and other genes) downregulation, thereby reducing cell proliferation and glycolysis. HIF-2α induces *Tie2* and *Ve-cadherin* expression, maintaining junctional integrity and promoting lymphangiogenesis.·O_2_^-^, radical superoxide anion; Arg arginine, Cit citrulline, CRCs chromatin remodeling complexes, DOT1L, DOT1-like histone lysine methyltransferase, eNOS endothelial nitric oxide synthase, GPX glutathione peroxidase, GR glutathione reductase H_2_O_2_, hydrogen peroxide, HIF hypoxia-inducible factor, miR microRNA, NO· nitric oxide, NOX NADPH oxidase, ONOO^-^ peroxynitrite, PRX peroxiredoxin, SOD superoxide dismutase. Figure was created with Adobe Illustrator and the DNA strand was created with BioRender.com.

Besides transcriptional and growth factors, emerging data have shown that other regulators such as miRNAs, epigenetics (Fig. 2) (Box 1), reactive and nitrogen oxygen species, hypoxia (Fig. 2) (Box 2), and metabolism (Section 3) all have pivotal roles in controlling lymphangiogenesis^58–61^.

In conclusion, established and emerging molecular regulators may cooperate with each other to control lymphangiogenesis, further revealing the complexity of the lymphatic system. While the relevance of VEGF-C/D/VEGFR-3 and other cytokine/receptor systems (FGF/FGFR-3, ANG2/TIE2) in lymphangiogenesis is indisputable, many other cytokines participate in the lymphatic regulation. They include the insulin-like growth factor (IGF-1), the hepatocyte growth factor (HGF), the platelet-derived growth factor B (PDGF-B), members belonging to the TGF-β superfamily, as wells as miRNAs, epigenetics, reactive and nitrogen oxygen species and hypoxia^38^. Thus, the fact that LECs are simultaneously regulated by multiple factors and undergo specific molecular programs in the different organs justifies the need to consider an integrated picture of their molecular regulation.

Box 1 Emerging molecular regulators of lymphatic gene expression **Role of miRNAs in the regulation of lymphangiogenesis**

Although cytokine/receptor pathways have traditionally been the most extensively studied lymphangiogenesis regulators by far, a plethora of studies conducted over the last decade have revealed a crucial role of miRNAs in both physiological and pathological settings. miRNAs are a group of endogenous non-coding RNAs of 19-25 nucleotides involved in the post-transcriptional gene regulation by complementary base-pairing with their target mRNAs^58^. These small molecules are modulators of multiple biological processes, including cell proliferation, redox homeostasis, apoptosis, differentiation, migration and invasion^153^. To date, the involvement of miRNAs in the lymphangiogenesis regulation constitutes one of the most active areas of research within the field.

Regarding physiological lymphangiogenesis, miR-31, miR-181a and miR-466 exert a negative regulation of VEC-to-LEC differentiation through direct binding to the 3’ untranslated (3’UTR) region of PROX1^154–156^ (Fig. 2A). Besides, miR-126 is involved in both in vitro and in vivo lymphatic development and tumor lymphangiogenesis through the positive regulation of LEC sprouting, proliferation and migration^157^. Specifically, miR-126 promotes the aforementioned processes by targeting two genes, PIK3R2 and SPRED1, which are involved in the PI3K/AKT and MAPK/ERK signaling pathways, respectively^158^ (Fig. 2A). Interestingly, miR-126 expression is regulated upstream by the Ang/Tie2 signaling axis, reinforcing the relevance of this cytokine/receptor system in lymphatic vessels and the complexity of the lymphatic regulation^157^ (Fig. 2A). Some differentially expressed miRNAs, including miR-128, miR-182-5p and miR-1236, have been ascribed to tumor-associated lymphangiogenesis in humans, exerting their functions by direct interference with the VEGF-C/VEGFR-3 axis^159–162^ (Fig. 2A).

The above examples highlight the important (and in some cases, crucial) role of miRNAs as molecular regulators of both developmental and pathological lymphangiogenesis. Although the interplay between miRNAs and metabolic gene expression has been described in multiple cell types, it is so far unknown whether miRNA-mediated regulation of lymphangiogenesis could play a role in lymphatic metabolic gene expression. Thus, further investigations are a pressing need to establish this link.

**Epigenetic regulation of lymphangiogenesis**

Beyond transcriptional and post-transcriptional networks, lymphatic growth regulation encompasses the epigenetic level, with a special involvement of histone acetylation, DNA methylation and chromatin-remodeling complexes^13,163,164^.

PROX1-FAO mediates epigenetic changes in LECs by histone acetylation of lymphangiogenic genes, and thus represent a crucial metabolism-dependent mechanism for LEC differentiation and the maintenance of lymphatic identity^13^. On the other hand, an interesting study conducted by Brönneke et al. in 2012 demonstrated that LECs and BECs differ in their DNA methylation patterns, and identified a role of DNA methylation in the regulation of lymphatic development and transdifferentiation^163^. Transdifferentiation processes imply the switch between BECs and LECs after genetic modification, and reflects the plasticity of these cell types. This phenomenon is present in LECs during early embryonic development and in pathological circumstances^4^. Importantly, through the analysis of global methylation and gene expression patterns, Brönneke et al. reported the existence of several differentially expressed and methylated genes in BECs and LECs, including PROX1, VEGFR-3 and its co-receptor NRP-2^163^ (Fig. 2B). The methylation of lymphangiogenic genes, including VEGF-C and Ang-2, has been associated with poor prognosis in melanoma patients and the methylation status has been proposed as a prognostic marker in these skin tumors^59^.

Furthermore, chromatin-remodeling complexes (CRC) exert an outstanding role in lymphatic development^164^. For instance, DOT1L (disruptor of telomeric silencing 1-like) is a H3K79 methyltransferase that binds LEC-specific genes, such as *Sox18* and *Flt4* and induce their transcriptional activation through chromatin relaxation^164^ (Fig. 2B). Hence, epigenetics is another important player to take into consideration to better understand the lymphatic regulation.

Box 2 Impact of oxygen and nitrogen species on lymphangiogenesis **Role of reactive oxygen and nitrogen species in LECs**

Although the function of reactive oxygen species (ROS) and nitric oxygen species (NOS) in lymphangiogenesis is still underexplored, a regulatory role has been already elucidated^165–167^. These chemical species result from the partial reduction of molecular oxygen. The three main types of ROS are: radical superoxide anion (·O_2_^-^), which is produced in the ETC or by NADPH oxidases (NOX); hydrogen peroxide (H_2_O_2_), which is generated by superoxide dismutases (SOD) or by spontaneous dismutation of ·O_2_^-^; and the hydroxyl radical (·OH), resulting from the reaction of H_2_O_2_ with Fe^2+^ (Fenton reaction). Moreover, after being produced, H_2_O_2_ can be reduced by action of either catalase, glutathione peroxidase (GPX)/glutathione reductase (GR) or peroxiredoxin (PRX) (Fig. 2C).

H_2_O_2_ can stimulate VEGF-C/VEGFR-3 pathway, further activating endothelial NOS (eNOS) and inducing nitric oxide (NO·) production (Fig. 2C). The latter has been associated with the induction of LEC proliferation; interestingly, a positive correlation between tumor NOS expression and lymphatic metastasis has been described^165–167^.

It is well known that ROS act as second messengers in multiple signaling cascades and are required for a wide range of biochemical processes^60^. Moreover, ROS play an important role in the maintenance of tissue homeostasis^60^. Considering the hormetic effect of ROS, which is harmful at high levels but essential for the correct performance of multiple biologic functions at moderate levels^168^, these findings may offer new avenues for the use of pro- and/or anti-oxidant strategies in the treatment of lymphatic-related diseases.

**Role of hypoxia in lymphatics**

An intriguing role of hypoxia in the regulation of (lymph)angiogenesis has been described^61,169,170^. During cancer progression, as the tumor grows, oxygen demands eventually outstrip the blood supply, resulting in the generation of a hypoxic microenvironment^63^. Although hypoxia is a positive regulator of (lymph)angiogenesis and promotes EC proliferation, a recent study has shown that hypoxia can also block LEC proliferation^61^. This effect is at least partly exerted by HIF-1α-mediated downregulation of the carcinoembryonic antigen-related cell adhesion molecule 1 (CEACAM1), protein that regulates LEC proliferation through the c-Jun N-terminal kinase (JNK) pathway^61^ (Fig. 2D). Furthermore, it is worth mentioning that HIF-1α has been conventionally linked to hypoxia, but its expression can be also upregulated in response to mechanical stimuli and shear stress^169^. Indeed, pulmonary lymph flow increases HIF-1α expression and decreases glycolysis in LECs^169^. This is a surprising fact given that HIF-1α has been previously described as a positive regulator of glycolytic enzymes^169^ (Fig. 2D). However, these seemingly incongruent results could be explained by the fact that LECs have a higher reliance on FAO than their blood counterparts, and therefore may exhibit specific metabolic adaptations to HIF-1α.

Importantly, Jiang and colleagues demonstrated that tissue hypoxia in lymphedema induces HIF-1α in LECs but reduces HIF-2α expression^170^. Conversely, HIF-2α upregulation restores lymphatic function and alleviates lymphedema. Mechanistically, HIF-2α regulates the ANG/TIE2 pathway in a positive way, consequently restoring the normal lymphatic function. Another downstream effect of ANG/TIE2 activation is VE-cadherin upregulation, which increases junctional integrity and helps improve the lymphatic drainage^170^ (Fig. 2D).

## Role of metabolism in the lymphatic vasculature

Upon the resurgence of focus on cancer cell metabolism, increasing attention has been paid to the role of metabolism in the function of immune and stromal cells in physiological and pathological contexts. For instance, numerous investigations have revealed that nutrient limitation in the TME triggers a situation in which cancer, stromal and immune cells must compete for the nutrients to carry out their biosynthetic and bioenergetic functions^62^. Indeed, the reprogramming of energy metabolism has been considered one of the emerging hallmarks of cancer in the last decade^63^. So far, the metabolism of cancer cells has been very well characterized, and we have known for nearly a century that tumor metabolism can differ markedly from healthy cell metabolism^63^. However, much less is known about the metabolism of infiltrating and stromal cells, such as endothelial cells, pericytes or fibroblasts.

Recently, the role of metabolism in the regulation of the blood endothelial cell (BEC) function has been elucidated^64–71^. In healthy adults, BECs remain quiescent but not hypometabolic. BECs still upregulate fatty acid oxidation (FAO) about 3-fold higher than proliferating activated cells, although for different purposes. While proliferating ECs rely on FAO to sustain the TCA cycle for biomass production, quiescent ECs prioritize maintaining an active TCA cycle to ensure redox homeostasis through NADPH production^70^.When quiescent BECs are activated, they become addicted to glucose^72^ and around 85% of the intracellular ATP is produced by glucose conversion to lactate^20^. In tumors, BECs display a hyperglycolytic phenotype when compared with healthy BECs, and there is a diversion of glycolytic intermediates to the pentose phosphate pathway (PPP) and serine biosynthesis pathways for nucleotide biosynthesis^20^. Supported by findings of the crucial role of metabolism in BEC regulation, metabolism of the lymphatic vasculature has recently emerged as a potential therapeutic approach and is being extensively studied to identify novel targets for treating lymphatic-related diseases^13,14,73^.

LECs exist in a unique milieu that may be partly responsible for their metabolic peculiarities, since the lymphatic endothelium is continuously exposed to high levels of glucose, proteins and fatty acids present within the lymph fluid^74,75^. Moreover, in stark contrast with the blood vasculature, where the oxygen concentration ranges between 80-100 mmHg, LECs are exposed to a relatively low oxygen concentration (8-35 mmHg)^76,77^.

It is evident that the molecular regulation of lymphangiogenesis has been extensively explored, but research on lymphatic metabolism has only recently taken off. However, the influence of LEC metabolism on lymphatic development, growth and function should not be underestimated since it has been already proven to be equally important in the regulation of both physiological and pathological lymphangiogenesis^11,13–17^. In this section, we provide a broad overview of the known metabolic pathways playing a crucial role in the lymphatic regulation.

### Fatty acid metabolism

#### Fatty acid β-oxidation and tricarboxylic acid cycle

**Fig. 3:**
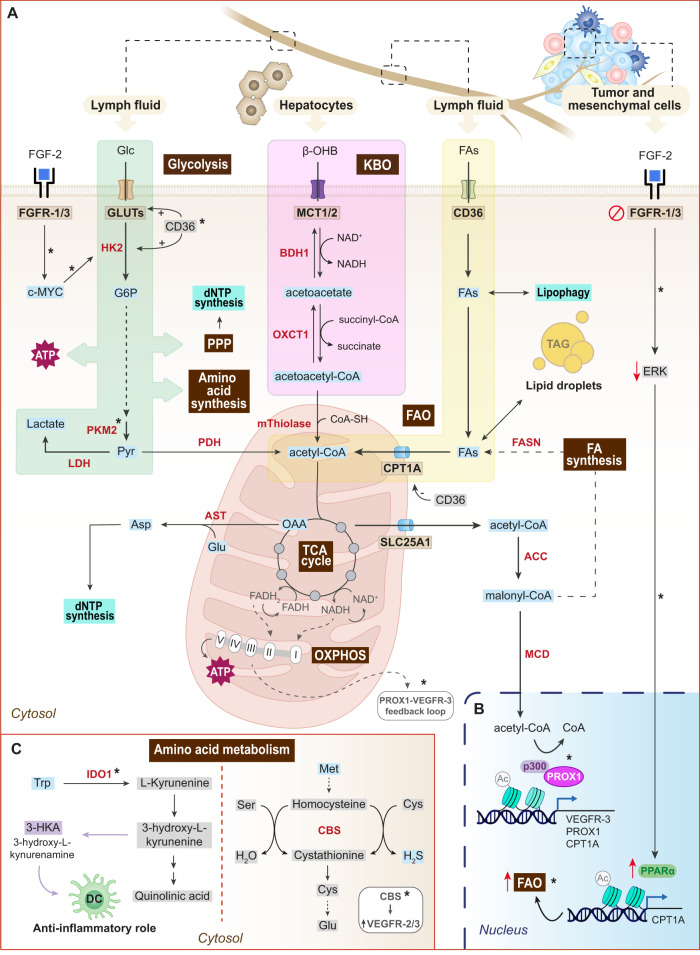
The metabolic engine of lymphatic endothelial cells. **A** Central metabolism. Lymphatic metabolism is primarily driven by fatty acid (FA) metabolism (yellow) and the tricarboxylic (TCA) cycle, ketone bodies oxidation (KBO) (pink) and glucose metabolism (green). FAs are incorporated from the lymph fluid via CD36 and are transported into the mitochondria through CPT1A. Acetyl-CoA produced by FAO enters the TCA cycle to be oxidized. Acetyl-CoA can also be diverted towards FA synthesis. FAs can be stored in lipid droplets in the form of triacylglycerols (TAG) and can be released via lipophagy. Oxalacetate obtained in the TCA cycle, together with glutamate, can produce aspartate (Asp), which contributes to dNTP synthesis. In KBO pathway, β-hydroxybutyrate (β-OHB) produced by hepatocytes can be incorporated by LECs, rendering acetoacetyl-CoA upon the consecutive reactions catalyzed by BDH1 and OXCT1 enzymes. In the mitochondria, acetoacetyl-CoA is converted into acetyl-CoA, which enters the TCA cycle. By producing acetyl-CoA, FAO and KBO support the TCA cycle and thereby contribute to dNTP synthesis and ATP production via oxidative phosphorylation (OXPHOS). Acetyl-CoA can be also transported to the cytosol through the SLC25A1 transporter, and then it can enter the nucleus. Moreover, malonyl-CoA produced during FA synthesis can be imported into the nucleus in the form of acetyl-CoA after decarboxylation by the malonyl-CoA decarboxylase (MCD). The complex III of the mitochondrial respiratory chain regulates the PROX1-VEGFR-3 feedback loop. Another important metabolic pathway in LECs is glycolysis. LECs incorporate glucose (Glc) from the lymph fluid and exhibit high glycolytic rates that contribute to ATP production, amino acid synthesis and pentose phosphate pathway (PPP). Moreover, upon interaction with FGFR-1/3, FGF-2 upregulates hexokinase-2 (HK2; rate-limiting enzyme of glycolysis) through the c-MYC transcription factor. **B** Transcriptional regulation of lymphatic metabolism. By acting as a p300 substrate, acetyl-CoA produced by FAO, in conjunction with PROX1, promotes lymphatic gene expression (VEGFR-3, PROX1 and CPT1A). Moreover, upon FGF/FGFR axis blockade, the impairment of the ERK signaling pathway triggers increased CPT1A expression through the activation of the nuclear receptor PPAR-α, finally promoting CPT1A transcription to stimulate FAO as a compensatory mechanism. **C** Amino acid metabolism. Tryptophan (Trp) metabolism involves the conversion of Trp to L-kyrunenine by indoleamine 2,3-dioxygenase (IDO1). L-kyrunenine can then be converted to 3-hydroxyl-L-kyrunenine, which can ultimately generate quinolinic acid (canonical pathway) or it can render the biogenic amine 3-hydroxy-L-kynurenamine (3-HKA). 3-HKA exerts an anti-inflammatory role by the activation of pro-inflammatory pathways within dendritic cells (DC). Cystathionine-β-synthase (CBS) modulates lymphatic function through the induction of VEGFR-2/−3 and the hydrogen sulfide (H2S) production during amino acids transulfurylation. This process involves the generation of cystathionine, H_2_S and water from methionine (Met)-derived homocysteine, cysteine (Cys) and serine (Ser). Further metabolization of cystathionine forms cysteine (Cys) and glutamate (Glu). ACC acetyl-CoA carboxylase, Asp aspartate AST aspartate aminotransferase, β-OHB β-hydroxybutyrate BDH1 D-β-hydroxybutyrate dehydrogenase, CBS cystathionine-β-synthase, Cys cysteine, DCs dendritic cells, FAO fatty acid oxidation, FAs fatty acids, FASN fatty acid synthase, G6P glucose-6-phosphate, Glu glutamate, GLUT glucose transporter, H_2_S hydrogen sulfide, HK2 hekoxinase-2, IDO1 indoleamine 2,3-dioxygenase, KBO ketone body oxidation, Lac lactate, LDH lactate dehydrogenase, MCD malonyl-CoA decarboxylase, MCT1/2 proton-linked monocarboxylate transporter, Met methionine, OAA oxalacetate, OXCT1 3-oxoacid CoA-transferase 1, OXPHOS oxidative phosphorylation, PDH pyruvate dehydrogenase, PPP pentose phosphate pathway, Pyr pyruvate, Ser serine, TAG triacylglycerols, TCA tricarboxylic acid, Trp tryptophan. The asterisks (*) indicate the unique molecular and metabolic peculiarities of the lymphatic endothelium. Figure was created with Adobe Illustrator and the DNA strand was created with BioRender.com.

Fatty acids (FA) are catabolized in the mitochondria through the FAO pathway, which has been described as a critical metabolic route in lymphatic vessel formation^13^. This process involves the breakdown of FAs into acetyl-CoA, which can be diverted to the TCA cycle, and the release of NADH and FADH_2_, which serve as electron donors for ATP production during OXPHOS (Fig. 3A)^73^.

Despite the well-established role of FAO in energy production in proliferative cells, pioneering work by Schoors et al. revealed that this metabolic pathway holds a unique role in the endothelium. This is explained by the fact that, unlike other cell types, the impact of FAO on energy generation and redox homeostasis is negligible in proliferating ECs^64^. Instead, FAO sustains the TCA cycle in conjunction with anaplerotic substrates, resulting in the production of the precursors glutamate and aspartate for de novo synthesis of purine and pyrimidine deoxynucleotides (dNTP) (Fig. 3A)^64^. Of note, in comparison with proliferating ECs, quiescent BECs upregulate FAO not to support biomass or energy production but to sustain the TCA cycle for redox homeostasis through NADPH regeneration^69^. This makes the endothelium unique in regard to FAO.

The metabolic preference of FAO-derived carbons towards dNTP synthesis has been validated in LECs, even proving to be markedly superior to that of BECs^13^. LECs exhibit a higher rate of FA uptake and oxidation compared with BECs, mainly due to the elevated expression of proteins involved in the binding, transport and import of these metabolites into the mitochondria, especially of the carnitine palmitoyltransferase 1 A (CPT1A)^2,13^. Known as the FAO rate-limiting enzyme, CPT1A is located in the outer mitochondrial membrane and is involved in the import of long-chain FAs for further β-oxidation and conversion into acyl-carnitines (Fig. 3A). Strikingly, a link between PROX1 transcriptional activity and FAO has been recently unveiled^13^. Using both in vitro and in vivo approaches, Wong and colleagues reported that PROX1 induces CPT1A expression in LECs, and the subsequent upregulation of FAO leads to increased acetyl-CoA levels. Acetyl-CoA serves as a donor substrate in the histone acetylation reaction catalyzed by p300^13^ (Fig. 3B). Acetylation of H3K9 in LEC-specific genes, including *FLT4*, results in enhanced chromatin decondensation, facilitating the access of PROX1 to these sites. Therefore, PROX1 uses FAO to promote its own transcriptional activity, while simultaneously creating a specific metabolic state crucial for the LEC differentiation and the maintenance of lymphatic identity^2,13,78^. This type of regulation has been recently recognized as the “epigenome-metabolome-epigenome” signaling cascade^79^. Despite the lack of further attempts to study PROX1-mediated transcriptional control of lymphatic metabolism, studies related to other PROX1-expressing tissues have shown that PROX1 modulates the expression of metabolic genes via a co-repressor/co-activator. For example, PROX1 is a co-repressor of LRH-1 (human liver receptor homolog-1) and suppresses the transcription of CYP7A1 (Cholesterol-7α-hydroxylase), the rate-limiting enzyme of bile acid synthesis^80^. In addition, PROX1 is a modulator of retinoic acid-related orphan receptors α- and γ-mediated transactivation^81^. Again, this evidence supports the implication of PROX1 in metabolic regulation.

Apart from PROX1, FAO metabolism in LECs interweaves with other cellular processes, such as autophagy. Autophagy is the main lysosomal pathway for intracellular disposal, degradation and recycling of cellular components, serving as a route to rapidly recycle metabolites that can be used for biosynthetic and/or energy production purposes. FAs are stored in lipid droplets in the form of triacylglycerol (TAG), and their degradation through autophagy (lipophagy) supplies FAs to the mitochondria^82^ (Fig. 3A). A recent study has shown that lipophagy fosters mitochondrial FAO through the degradation of lipid droplets in LECs, and thereby, maintains acetyl-CoA levels to sustain CPT1A expression and H3K9 acetylation, resulting in the epigenetic regulation of PROX1-target genes^83^. Conversely, loss of LEC autophagy impairs VEGFR-3 expression and affects mitochondrial dynamics^83^. These findings highlight a role of lipophagy in the lymphatic FAO and TCA cycle, which are required for the maintenance of the PROX1-VEGFR3 feedback loop, metabolic homeostasis and lymphatic identity^2,83^ (Fig. 3A).

Aside from assisting in histone acetylation, FA-derived acetyl-CoA is essential for biomass production, LEC proliferation, migration and sprouting since it sustains the TCA cycle (along with other anaplerotic substrates) and promotes dNTP synthesis. This makes FAO the main metabolic pathway controlling lymphangiogenesis, and may also explain why VEGF-C/VEGFR-3 increases FAO flux in order to support lymphangiogenesis in adult tissues^2^.

#### FA synthesis

Besides FAO, FA synthesis is also crucial for lymphangiogenesis^15^. This anabolic pathway involves the generation of FAs from acetyl-CoA, which first needs to be carboxylated to yield malonyl-CoA in a reaction catalyzed by the acetyl-CoA carboxylase. Following condensation and reduction of malonyl-CoA building blocks, palmitate is generated in a reaction catalyzed by the fatty acid synthase (FASN), the rate-limiting enzyme of FA synthesis (Fig. 3A). Interestingly, pharmacological inhibition of the FA synthase (FASN) with cerulenin and orlistat in LECs is associated with decreased cell viability, proliferation and migration in vitro and anti-metastatic effects in vivo^15^. B16-F10 melanoma-bearing mice treated with orlistat showed a reduction in the metastatic lymph node (LN) burden, as well as decreased VEGF-C and VEGFR-3 expression levels, thereby promoting an anti-lymphangiogenic phenotype^15^. These data support the role of FA synthesis in lymphangiogenesis, but additional studies are required to elucidate the exact molecular mechanism.

### Ketone bodies oxidation pathway

Although FAO is an important source of acetyl-CoA, it does not stand alone. The other main source of acetyl-CoA are ketone bodies. Ketone bodies are energy-rich metabolites produced by hepatocytes and yield two molecules of acetyl-CoA when oxidated extrahepatically^84^. LECs have access to ketone bodies and are able to oxidize them to acetyl-CoA through the KBO (Fig. 3A). To fully determine the impact of KBO on LECs, lentiviral vectors were used to silence the 3-oxoacid-CoA-transferase-1 (OXCT1) expression, the KBO rate-controlling enzyme^16^. As a result, a reduction of acetyl-CoA levels to sustain the TCA cycle and nucleotide synthesis, along with reduced LEC proliferation, migration and sprouting, was observed. Similarly, cardiac ECs are able to oxidize ketone bodies and this enhances cell proliferation, migration and vessel sprouting^85^. In vivo, LEC-specific loss of OXCT1 impaired lymphatic growth in developmental and pathological models. On the contrary, ketone bodies supplementation increased lymphangiogenesis in both LEC cultures and murine models^16^. Although a possible epigenetic-mediated gene expression mechanism was proposed based on the observations that OXCT1 silencing in LECs increased H3K27me levels, the epigenetic effect of ketone bodies in LECs needs additional investigations^16^. Interestingly, the results obtained in a mouse model of microsurgical ablation of lymphatic vessels in the tail, which recapitulates features of acquired lymphedema in humans, suggested an intriguing range of potential nutritional and metabolite-based approaches to treat primary or more common secondary lymphedema in humans^16^.

### Glucose metabolism

Both angiogenesis and lymphangiogenesis involve endothelial cell proliferation, migration, and sprouting, which are all energy-dependent processes. Contrary to the remarkable difference in FAO rates between proliferating BECs and LECs, both cell types are highly glycolytic and rely on this metabolic pathway for ATP production during proliferation and migration^73^. However, FAO is only involved in proliferation through biomass production, rather than ATP generation^64^. This phenomenon reveals distinct metabolic requirements for the two main activities of ECs during (lymph)angiogenesis, proliferation (requires glycolysis and FAO) and migration (requires glycolysis but not FAO). To sustain proliferation and migration, LECs rely on anaerobic glycolysis as their main energy source, since it produces more than 70% of the total ATP. This is not surprising considering that oxygen concentration within the lymphatic environment is relatively low^73^. In comparison with the complete oxidation of glucose through OXPHOS, which renders 32 ATP molecules per mole of glucose, anaerobic glycolysis has a net production of 2 moles of ATP per mole of glucose during the conversion of glucose to lactate. Despite the apparent inefficiency of anaerobic glycolysis, this bioenergetic setting has already been shown to be considerably advantageous. Firstly, maintaining a sufficiently high glycolytic flux may allow for abundant ATP production in a fast manner, further enabling LECs to rapidly respond to the increased energetic demands during lymphangiogenesis. In fact, the amount of ATP produced during glycolysis may even exceed that of OXPHOS if the glycolytic flux is sufficiently high^86^. Secondly, as described in cancer cells, an elevated glycolytic flux may also help maintain complete pools of glycolytic intermediate metabolites. This would favor the reconduction of the metabolic flux towards the PPP and other non-mitochondrial biosynthetic pathways branching off from glycolysis, including the synthesis of lipids, serine, nucleotides and amino acids^67,86,87^ (Fig. 3A). Thirdly, relying on anaerobic glycolysis does not only lower the generation of ROS, but also allows for the generation of reductive power in the PPP, thereby assuring the maintenance of redox homeostasis^14,88^. Hence, although anaerobic glycolysis may seem an apparently inefficient pathway in terms of ATP production, it could provide LECs with numerous bioenergetic, biosynthetic and redox modulatory benefits.

FGF/FGFR signaling pathway promotes the glycolytic flux through the activation of c-MYC transcription factor, which subsequently induces the expression of the hexokinase-2 (HK2), the first glycolysis rate-controlling enzyme (Fig. 3A). Thus, in the absence of FGF signaling, decreased HK2 levels lead to reduced glycolysis and impaired endothelial cell proliferation and migration. This phenomenon has been described both during embryonic development and in adult tissue lymphangiogenesis^14,89^. Notwithstanding, the effect of HK2 silencing on lymphangiogenesis may not be entirely associated to ATP generation, as this enzyme regulates additional metabolic pathways associated to glycolysis, including glycosylation and PPP^73^. Recent evidence has shown that, if the FGF/FGFR axis is blocked and thus glycolytic flux is reduced, FAO would increase as a compensatory mechanism through the induction of CPT1A expression. Mechanistically, this metabolic flexibility is mediated by ERK signaling pathway, since FGFR blockade impairs ERK leading to increased CPT1A expression through the activation of the nuclear receptor PPAR-α^90^ (Fig. 3A, B). From a functional point of view, the disruption of the balance between glycolysis and FAO undoubtedly affects energy production as well as cellular proliferation and migration. Given that the FGF/FGFR signaling axis is considered one of the main lymphangiogenesis inductors, strategies targeting this complex are currently under development. Due to FAO compensatory upregulation, approaches against FGFR would likely be more successful if they simultaneously target FAO as well^90^.

Novel mediators of glycolysis have been lately discovered in LECs^91,92^. For instance, pyruvate kinase M2 (PKM2), which catalyzes the final and irreversible step of glycolysis to generate pyruvate and ATP (Fig. 3A), controls LEC proliferation, migration, tube formation and invasion^91^. Interestingly, high PKM2 expression has been detected in lymphatic vessels of lymphatic malformations, and congenital anomalies arising from abnormal and disorganized lymphatic vessels in the embryo^91^. These results show the significant role of PKM2 in lymphangiogenesis as a glycolysis regulator, and suggest that it could be used as a therapeutic target^91^. Furthermore, the fatty acid transporter CD36 has emerged as a dual FAO and glycolysis regulator in intestinal lymphatics^92^. In fact, its silencing inhibited FAO by downregulation of two key FAO enzymes, CPT1A and ACSL1 (acyl-CoA synthetase long-chain family member 1) and increased glycolytic rates by upregulation of glycolytic genes, including HK2 and GLUT1 (glucose transporter 1). These gene expression reductions are attributed to reduced VEGF-C mediated VEGFR-2/AKT signaling, and the impaired signaling pathway affect LEC migration, tube formation, and monolayer integrity^92^. In vivo, inducible CD36 deletion in LECs caused leaky intestinal lymphatic vessels, accumulation of inflamed visceral adipose tissue, and spontaneous late-onset obesity^92^. Thus, similar to PROX1-VEGFR-3 regulation, FAO-VEGFR-2/AKT connection highlights another interplay between two regulatory levels, molecular and metabolic, further demonstrating the complexity of the LEC regulatory network.

Moreover, considering glucose metabolism in LECs, it is worth highlighting an elegant metabolomics study describing that breast cancer cells induce LEC metabolic reprogramming in vitro. A total of 12 metabolic pathways were found to be significantly altered in LECs co-cultured with breast cancer cells, including an enrichment in glycolysis and pyruvate metabolism^93^. Although an increased dependence on glycolysis was observed, ROS levels were reduced, presumably showing that LECs might increase their antioxidant levels as a mechanism to maintain redox homeostasis. Lactate levels were significantly increased and correlated to lactate metabolism upregulation, which is in agreement with the higher glycolytic rate described^93^. This pioneering study reveals the ability of cancer cells to reprogram lymphatic metabolism in vitro and suggests that LECs undergo metabolic reprogramming during cancer progression in vivo.

### Oxidative phosphorylation

As stated before, the PROX1-VEGFR-3 positive feedback loop determines the number of LECs that originate from venous precursors and the maintenance of LEC fate^94^. This regulatory process seems to be controlled by the complex III of the Electron Transport Chain (ETC). During mitochondrial respiration, NADH and FADH_2_ generated in the TCA cycle donate electrons to the ETC, and this electron flow finally generates the membrane potential required for the ATP production^88^ (Fig. 3A). Recently, Ma and colleagues have unraveled that mitochondrial complex III can sense the LEC differentiation status and the metabolic conditions and, in response, regulates *Flt4* and *Prox1* expression^17^. In fact, deletion of the QPC subunit of mitochondrial complex III in differentiating LECs leads to mice devoid of lymphatic vasculature by mid-gestation^17^. This phenotype is due to reduced H3K4Me3 and H3K27ac in the promoter region of key LEC fate regulators, particularly *Flt4*. This would result in decreased VEGFR-3 expression, which would disturb the critical PROX1-VEGFR-3 feedback loop and result in a reduction of the number of lymphatic precursors. Simultaneously, this phenotype could be enhanced through other elements involved in the lymphatic development regulation, such as CPT1A^17^. In conclusion, mitochondrial complex III is required in LECs to properly maintain the PROX1-VEGFR-3 autoregulatory feedback loop, and therefore, LEC fate specification and maintenance.

The significance of the ETC complex III has also been described in BECs during angiogenesis^95^. Diebold et al. showed that inhibition of the mitochondrial complex III reduced EC proliferation, but not migration, by decreasing the NAD^+^/NADH ratio^95^. Since BECs rely on glycolysis as their main energy source, it is not likely that this effect was due to reduced ATP production. Nevertheless, the proliferation defect and impaired in vitro and in vivo angiogenesis observed by these authors could be explained by the inability of BECs to generate metabolic intermediates for biosynthetic purposes.

### Amino acid metabolism

Amino acid metabolism is important for energy homeostasis in multiple cell types^66,67,70^. However, most research has focused on BECs^66,67,70^, and reports showing the significance of amino acid metabolism in LECs are still scarce. One of the studies on the role of amino acids in LECs comes from Clement and colleagues, who link tryptophan catabolism to the immunological profile of LECs^18^. These authors described the role of 3-hydroxykynurenine (3-HKA), a biogenic amine that is produced in not only dendritic cells but also at high levels in LECs and tumor cells, and is generated through an alternative pathway of tryptophan metabolism^18^. Interestingly, deletion of IDO1, the rate-limiting enzyme of 3-HKA production, increased skin inflammation and exacerbated psoriasis in mice. This pathology was at least partly due to the activation of the pro-inflammatory IFN-γ mediated STAT1/NF-κΒ pathway^18^. Therefore, LECs might use 3-HKA to exert an anti-inflammatory role with the consequent decrease in the release of pro-inflammatory chemokines and cytokines (Fig. 3C).

Another study has unveiled a role for cystathionine β-synthase (CBS) in regulating lymphangiogenesis^96^. This enzyme is involved in the tran-sulfurylation of sulfur amino acids (i.e., methionine, cysteine, homocysteine, and taurine) and the production of H_2_S, which has been previously reported as a pro-angiogenic factor^97^. Upon CBS blockade, LEC proliferation, migration, and tube formation were all reduced. Interestingly, CBS inhibition also led to a decrease in VEGFR-2 and VEGFR-3 expression, thereby suggesting that it could be contemplated as a new therapeutic target^96^ (Fig. 3C).

Hence, studies over the last decade have revealed the importance of LEC metabolism in both developmental and adult lymphangiogenesis, further depicting that this metabolic modulation can override the instructions of lymphangiogenic signals. Although it is widely accepted that lymphatic function is tightly regulated at multiple levels, further efforts are needed to better describe how molecular players intertwined with the metabolic networks. Meanwhile, despite several similarities between LECs and BECs, these two cell types exist in different environments and have generally divergent regulatory and metabolic networks. Therefore, findings related to the blood vasculature should not be extrapolated to the lymphatic endothelium without further investigation, although they may provide clues for building pillars to help guide lymphangiogenesis research.

## Role of lymphatic vessels in pathology

Impaired lymphangiogenesis or lymphatic dysfunction contributes to the progression of several pathologies, including lymphedema, neurological and neurodegenerative disorders, inflammatory bowel disease, cardiovascular disease, metabolic syndrome, and glaucoma. On the other hand, inflammation and cancer are linked to excess lymphangiogenesis^1^. Understanding lymphatic function in disease is an unmet clinical need for the development of novel and more specific pro- and anti-lymphangiogenic therapeutic strategies. In the next subsections, the main lymphatic-related diseases are described.

### Cancer

The connection between lymphatic vessels and tumor biology was established at the beginning of the 21st century and has been an area of ongoing research ever since. The permeable structure of lymphatic vessels and the high interstitial pressure within solid tumors favor cancer cell intravasation. Furthermore, lymphangiogenesis provides a physical pathway for metastatic dissemination towards LNs, and ultimately to distant organs^98^. It is well known that dissemination of many types of cancer, such as melanoma, breast, oral, pancreatic, and cervical cancers, occurs preferentially through the lymphatic system^99^. In these types of cancer, LNs are the first sites to be invaded. Indeed, the presence or absence of cancer cells in the first draining LN (also known as sentinel LN) has been recognized as a prognostic factor in several human cancers, often being even more reliable than already established clinical parameters such as tumor size or histological grade^100,101^.

Although there is undisputed evidence to support the connection between lymphangiogenesis and the promotion of LN metastases, the underlying mechanisms and relationship between tumor-associated lymphatic vessels and cancer cell dissemination to peripheral organs are incompletely understood, despite the agreement that the presence of LN metastases is a robust indicator of cancer progression, aggressiveness, and overall cancer cell disseminative capacity^98^.

**Fig. 4:**
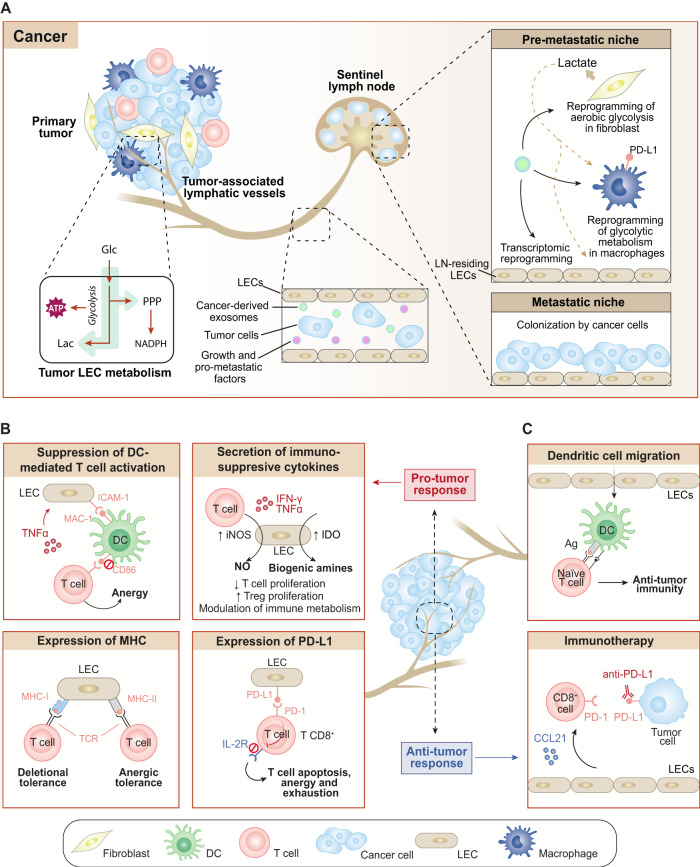
Role of tumor-associated lymphatic vessels. **A** Lymphatic vessels are conduits for cancer cell dissemination from primary tumors to sentinel LNs. Tumor LECs undergo metabolic reprogramming towards increased glucose consumption and glycolytic rates. Besides, LN-residing LECs undergo transcriptional reprogramming, initially in response to cancer-derived exosomes and growth factors (pre-metastatic niche), and eventually by direct interaction with cancer cells (metastatic niche). Cancer-derived exosomes also induce metabolic reprogramming of other LN-residing cell types, such as fibroblasts and macrophages. The metabolic reprogramming of fibroblasts towards increased aerobic glycolysis leads to the release of lactate, which can be incorporated and used as energy fuels by other cell types, such as macrophages and LECs. Tumor-derived exosomes can also act on macrophages inducing their glycolytic metabolism reprogramming and promoting an immunosuppressive phenotype characterized by PD-L1 upregulation. **B**, **C** Dual role of lymphatics in immunity. **B** LECs evoke a pro-tumor immune response through different mechanisms: i) the suppression of DC-mediated T cell activation via MAC-1/ICAM-1 interactions in response to TNF-α and the consequent CD86 blockade in DCs, which induces T cell anergy; ii) in response to IFN-γ and TNF-α, LECs secrete nitric oxide (NO) and biogenic amines with immunosuppressive activities; iii) expression of MHC-I and MHC-II molecules in LECs induce deletional and anergic T-cell tolerance upon interaction with T cell receptors (TCR); and iv) PD-L1 expression in LECs is also associated to a pro-tumor response, since it blocks the IL-2R receptor in T cells upon interaction with PD-1, inducing T cell apoptosis, energy and exhaustion. **C** Conversely, LECs exhibit anti-tumor properties through the induction of DC migration towards LNs and the secretion of immunosuppressive cytokines. In response to CCL21, LECs also support the recruitment of naïve T cells, which express PD-1 molecules. Interestingly, lymphangiogenesis has improved the outcome in cancers by facilitating the access of anti-PD-L1 antibodies used in immunotherapy. Ag, antigen; DC, dendritic cell; Glc, glucose; IDO, indoleamine 2,3-dioxygenase; IFN-γ, interferon-γ; IL-2R, interleukin-2 receptor; iNOS, inducible nitric oxide synthase; Lac, lactate; MHC, major histocompatibility complex; NO, nitric oxide; PD-(L)1, programmed death protein (ligand) 1; PPP, pentose phosphate pathway; TCR, T cell receptor; TNF-α, tumor necrosis factor α. The figure was created with Adobe Illustrator.

Interestingly, LN microenvironment must first be remodeled by cancer cells to become a supportive metastatic niche^99^. A pioneering study conducted by Kaplan et al. introduced the concept of ‘pre-metastatic niche’, which refers to the preparation of the LN microenvironment before tumor cell arrival^102^. Cancer cells present within the primary tumor release pro-metastatic factors and extracellular vesicles that facilitate the hosting of tumor cells in the LNs^99,102^ (Fig. 4A). In this context, the remodeling of the LN microenvironment during tumor progression likely involves reprogramming of resident cell types. Indeed, tumor-derived extracellular vesicles are able to reprogram macrophage glycolytic metabolism in the pre-metastatic niche, upregulating PD-L1 and acquiring an immunosuppressive phenotype^103^ (Fig. 4A). The stromal fibroblast metabolism is also reprogrammed towards increased aerobic glycolysis by melanoma exosome miRNAs^104^. Increased glycolysis in fibroblasts might be interpreted as a mechanism to produce high energy fuels (such as lactate) that could be used by other cell types within the LN microenvironment (Fig. 4A). Furthermore, it has recently been elucidated that melanoma-derived exosomes can induce transcriptional changes in LECs within LNs, including upregulation of genes with potential immunomodulatory functions^105^ (Fig. 4A).

An important concept related to tumor-associated lymphatics is their dual role in tumor immunity, as they can be involved in pro-tumor, but also in anti-tumor immune responses (Fig. 4B, C). Lymphatics might not only promote tumor progression via enhanced metastasis, but also through contribution to the establishment and maintenance of an immunosuppressive tumor microenvironment^1,106^. However, a positive correlation between lymphatic vessel density and anti-tumor immunity has been already reported, thereby supporting the role of lymphatics in immunosurveillance^106,107^.

Regarding LEC pro-tumor responses, accumulated evidence has shown that they rely on T cell tolerance induction, which is mediated by four major mechanisms^108–112^ (Fig. 4B): i) suppression of dendritic cell-mediated T cell activation via MAC-1/ICAM-1-mediated inhibition of CD86, which eventually leads to T cell anergy^108^; ii) secretion of immunosuppressive cytokines (i.e., NO and biogenic amines) in response to IFN-γ and TNF-α^109,110^; iii) expression of MHC class I and II molecules, thereby inducing T cell deletional and anergy, respectively^111,113^; and iv) expression of PD-L1 and lack of co-stimulation leading to high-level of PD-1 expression on CD8^+^ T cells and IL-2R inhibition^112^. Negative signals induced through PD-L1 signaling in activated T cells promote T-cell apoptosis, anergy and contribute to the progressive loss of T cell function (T cell exhaustion)^114^. In line with the fact that PD-L1 expression in LECs contributes to peripheral tolerance by limiting T cell activation, it has been shown that inhibition of PD-L1 expression in the endothelium triggers autoimmune reactions^112^. In addition, certain cell types reprogram their metabolic network in tumor draining LNs to acquire an immunosuppressive phenotype^106,115^.

On the contrary, the anti-tumor effects are partly mediated by the induction of dendritic cell migration towards tumor-draining LNs and the consequent DC-mediated antigen presentation to naïve T cells (Fig. 4C)^106,107^. Besides, tumor-associated LECs seem to have a role in potentiating immunotherapy through the recruitment of naïve T cells via secretion of CCL21, which is induced in response to VEGF-C^116^. Importantly, the aggressiveness of brain tumors relies mainly on the phenomenon of the immune privilege of the central nervous system (CNS), which describes the attenuated immune reactions in the brain as a result of limited penetration of the blood brain barrier by immune cells and other physiological characteristics of the CNS, such as low levels of major histocompatibility complex (MHC) class I and II molecules^62^. The existence of CNS immune privilege may impede checkpoint inhibitors to reach the brain and access the tumor. In agreement with this possibility, a recent study has revealed VEGF-C stimulated lymphangiogenesis to facilitate the access of anti-PD-1/CTLA-4 antibodies to the brain, thereby reducing the progression of brain tumors^117^. Thus, although the inhibition of lymphangiogenesis has conventionally been considered the main strategy to treat cancer, these findings show that extrapolation is not plausible within different cancer types and spatial heterogeneity must be also considered.

Although the VEGF-C/D/VEGFR-3 signaling axis has been the most widely studied for anti-lymphangiogenic therapies in cancer, the success of VEGF(R)-based therapies is restricted by intrinsic refractoriness and only modest efficacy in the context of cancer^20^. Therefore, the limitations of VEGF(R) blockade, together with the existence of alternative pathways controlling lymphangiogenesis, justify the need to deepen our understanding of tumor LEC metabolism. Indeed, acting at the metabolic level has been proposed as a novel approach to treat tumor (lymph)angiogenesis without causing systemic toxicity^118^. Rather than killing tumor ECs, tumor vessel normalization aims to restore their endothelial metabolism to that of healthy ECs^65^. In the context of tumor lymphatics, expanding our knowledge on the metabolic bases underlaying lymphatic modulation offers a promising therapeutic opportunity.

### Lymphedema

**Fig. 5:**
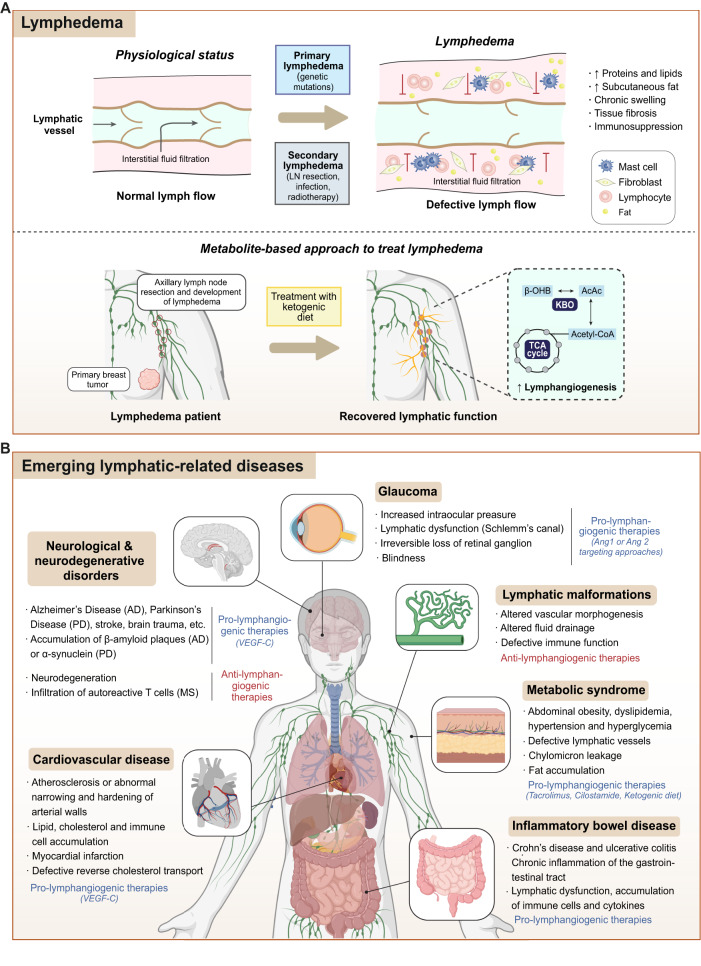
Lymphatic vessels in lymphedema and other lymphatic-related pathologies. **A** Lymphedema. Defective lymph flow during primary or secondary lymphedema disrupts interstitial fluid filtration and induces accumulation of proteins, lipids and fat, which promote chronic swelling, tissue fibrosis, and immunosuppression. Treatment with a ketogenic diet has been proposed as a metabolite-based approach to improve lymphedema. Elevation of lymph ketone body levels by a high-fat, low-carbohydrate ketogenic diet increases acetyl-CoA levels, thus inducing lymphangiogenesis and the recovery of the lymphatic function. **B** Lymphatics-related diseases. Lymphatics are in the scope of other diseases, such as neurological and neurodegenerative disorders (i.e., Alzheimer’s and Parkinson’s disease, stroke and brain trauma), cardiovascular disease (i.e., atherosclerosis and myocardial infarction), glaucoma, lymphatic malformations, metabolic syndrome and inflammatory bowel disease (i.e., Crohn’s disease and ulcerative colitis). AcAc, acetoacetate; AD, Alzheimer’s disease; IBD, intestinal bowel disease; MS, multiple sclerosis; PD, Parkinson’s disease; TCA, tricarboxylic acid cycle; β-OHB, β-hydroxybutyrate. Figure was created with Adobe Illustrator. Human body illustration in Fig. 5A and Fig. 5B were created with BioRender.com.

Disturbed lymphatic drainage following lymphatic dysfunction and defective lymph flow can result in lymphedema. This disease is referred to as primary lymphedema when it originates from genetic mutations, and secondary lymphedema when the lymphatic disruption appears in a non-genetic context^119^. A cause for the development of secondary lymphedema includes the removal of LNs following cancer treatment to prevent metastasis, especially in breast cancer, melanoma and gynecologic cancers^120^. Although LN resection is an essential precaution, it can damage the lymphatic vasculature, triggering the appearance of lymphedema in a considerable percentage of patients, oscillating between 14 and 40 % after breast cancer surgery, for instance^119^. In addition, secondary lymphedema may also develop after infection (as in filariasis) or radiotherapy^1^. Independent of its origin, impaired lymph absorption results in the accumulation of proteins and lipids in the interstitial space, chronic swelling (edema), tissue fibrosis, subcutaneous fat accumulation and immunosuppression^121^ (Fig. 5A). Despite its medical and social importance, no approved pharmacological treatment is available, and only a symptom-controlling physiotherapy exists^119^. Induction of lymphangiogenesis appears to be a suitable therapeutic strategy for the treatment of this disease. In fact, based on preclinical data about the pro-lymphangiogenic potential of ketone bodies, the phase II clinical trial Ketolymph (ref. NCT03991897) has evaluated the therapeutic potential of the ketogenic diet to reduce lymphedema^16^ (Fig. 5A). This illustrates how a metabolite-based approach can alleviate human lymphedema by increasing the formation and function of lymphatic vessels.

### Neurological and neurodegenerative disorders

Concerning neurological and neurodegenerative disorders, the emerging concept of the glymphatic system, a “pseudo-lymphatic” perivascular network distributed throughout the brain, and responsible for replenishing as well as cleansing the brain^122^, has replaced the longstanding dogma that the central nervous system (CNS) lacked lymphatic vasculature^122^. The brain is enveloped within three membranous layers of connective tissue, called the meninges, and functional lymphatic vessels are present within the outermost layer, the dura matter^123^. Meningeal lymphatics drain cerebrospinal fluid to deep cervical LNs, and they have been linked to the pathogenesis of a number of neurological and neurodegenerative diseases, including Alzheimer’s disease (AD), Parkinson’s disease (PD), stroke and brain trauma^123^. This is not surprising considering that these pathologies are characterized by immune dysfunction and the accumulation of aggregates (β-amyloid plaques and abnormal tau in AD, and α-synuclein in PD), which may potentially be cleared by the lymphatic system (Fig. 5B). In fact, disruption of meningeal lymphatic vessels increases β-amyloid and α-synuclein aggregate levels in mouse models of AD and PD^124^, and induces neuroinflammation in the latter^125^. In agreement, VEGF-C-mediated induction of meningeal lymphangiogenesis enhances glymphatic perfusion and improves the outcome of AD in these mouse models^124^. In contrast with the ameliorating effect of lymphatic vessels in these diseases, ablation of meningeal lymphatics inhibits the progression of multiple sclerosis^126^. In this autoimmune demyelinating disease, meningeal lymphatics support the T cell infiltration in the CNS^123^ (Fig. 5B). Therefore, the context and disease-dependent response to lymphangiogenesis/lymphatic ablation must be very closely studied to develop therapeutic strategies targeting the specific neurological disease.

### Inflammatory bowel disease

The intestinal lymphatic system is mainly comprised by two types of vessels: lacteals, present within the intestinal villi, and mesenteric collecting lymphatic vessels. These vessels play a pivotal role in the absorption of dietary lipids, reverse cholesterol transport and immune responses^127^. Therefore, functional and structural alterations of lymphatic vessels are frequent findings in patients with inflammatory bowel disease (IBD), which includes Crohn’s disease and ulcerative colitis^127^. IBD is mainly driven by a combination of genetic determinants and exposure to environmental risk factors. In patients with IBD, lymphatic dysfunction results in chronic inflammation of the gastrointestinal tract due to the accumulation of immune cells and cytokines, and the impairment of the fluid balance^128^ (Fig. 5B).

### Cardiovascular disease

Lymphatic vessels are present in all layers of the human heart and their functions include the maintenance of the tissue’s fluid homeostasis and immunosurveillance^129^. Although the presence of cardiac lymphatic vessels was reported more than 100 years ago^130^, their functional role in disease has not been addressed until the last decade, when a link between lymphatics, atherosclerosis, and myocardial infarction has been established^131^. Atherosclerosis is characterized by abnormal narrowing and hardening of arterial walls because of the lipid, cholesterol, and immune cell accumulation, which impedes blood flow (Fig. 5B). In the atherosclerotic plaques, a defective lymphangiogenesis with a consequent lipid accumulation, T cell enrichment and increased lesion formation has been detected^132^. Therefore, induction of lymphangiogenesis is a plausible strategy against this disease. This is supported by studies showing that VEGF-C-mediated lymphangiogenesis promotes cholesterol removal from atherosclerotic arteries and improves reverse cholesterol transport, thereby ameliorating the outcome of this disease^133,134^ (Fig. 5B). Furthermore, induction of lymphangiogenesis has a positive effect in post-myocardial infarction recovery, since clearance of fluid and debris by lymphatic vessels, along with immune cell extravasation, facilitate tissue remodeling and wound healing^44,129^.

### Metabolic syndrome

Metabolic syndrome is a pathological condition defined by abdominal obesity, dyslipidemia, hypertension, and hyperglycemia^135^ (Fig. 5B). From the point of view of systemic metabolism, obesity is associated with lymphatic defects, as demonstrated in experiments performed in *Prox1* + */-* obese mice with defective lymphatics, displaying chylomicron leakage into the mesenteric tissue and consequent fat accumulation^136^. In agreement, methods to stimulate lymphangiogenesis have become attractive to treat metabolic syndrome (Fig. 5B). For example, the administration of tacrolimus (drug able to improve lymphatic transport and reduce inflammation)^137^, or cilostamide (a phosphodiesterase3 inhibitor that reduces lymphatic permeability)^138^, have been proposed for the treatment of metabolic syndrome. Moreover, the uptake of a high-fat, low-carbohydrate ketogenic diet, which increases β-hydroxybutyrate circulating levels, induces lymphangiogenesis and improves weight loss for possible amelioration of metabolic syndromes^139^.

### Glaucoma

In physiological conditions, the intraocular pressure is regulated by the balance between the production and removal of the aqueous humor. The main cause of glaucoma is chronic elevated intraocular pressure due to the impaired aqueous humor outflow, which eventually leads to irreversible loss of retinal ganglion and blindness^140^ (Fig. 5B). The Schlemm’s canal (SC) is an endothelium-lined vascular channel in the eye that shares molecular and morphological features of both BECs and LECs, and maintains intraocular fluid homeostasis by draining aqueous humor into the systemic circulation^140^. Lymphatic dysfunction in the SC has been associated with ocular hypertension and glaucoma progression^141,142^. Indeed, the Ang1/Ang2-Tie2 axis plays a pivotal role in the maintenance of intraocular pressure, since knockout mice for *Ang1* or *Ang2* displayed impaired aqueous humor drainage and developed glaucoma^141,142^. Similarities between SC and lymphatic vessels raise the possibility of targeting glaucoma via pro-lymphangiogenic therapies.

### Lymphatic malformations

Vascular malformations refer to congenital alterations in vascular morphogenesis. Among them, lymphatic malformations (LM) comprises 10% and often alter fluid drainage and promote primary lymphedema and defective immune function^143^. Although surgery, sclerotherapy, and laser treatment have contributed to advances in the treatment of LMs, high recurrence rates are still an obstacle^144^. Research on LM metabolism has found that somatic mutations in the PIK3CA gene in LECs cause increased PI3K activity and phosphorylation of AKT, which contributes to increased LEC proliferation and lymphangiogenesis^145^. Recent studies have also found that PIK3CA-driven mutations have increased VEGFR3 signaling and that inhibition of VEGF-C signaling reduces LMs^146^. The glycolytic enzyme PKM2 is also augmented in LM tissues, where PKM2 contributes to the formation of LM lesions and correlates with infection of LMs, therefore establishing a connection between LEC metabolism, LMs and inflammation^144^. Therefore, targeting LEC metabolism is starting to emerge as an appealing pharmacological strategy to treat LMs (Fig. 5B).

There has been outstanding progress towards the identification of lymphatic dysfunction in different pathological conditions. Lymphatic diseases are recurrent and diverse, mainly due to the heterogeneous roles played by lymphatic vessels in different tissues and microenvironments^1^. Interestingly, these pathologies not only have the lymphatic system as a source, but also as a potential treatment-mediator, since lymphatics are an ideal route for drug transport and delivery. Most of the strategies tested so far have focused on either reactivating or deactivating lymphangiogenesis via targeting the VEGF-C/D/VEGFR3 axis. Adrenomedullin, a bioactive peptide involved in lymphatic function and regeneration, has also shown promising results in the development of lymphatic therapeutics^72,147^. However, treatment of lymphatic diseases is still scarce.

Ongoing research will strengthen our understanding of the lymphatic system and will launch the development of novel therapeutic strategies in the near future. The design of effective treatments would require a better understanding of lymphatic metabolism and signaling pathways, altogether with the identification of reliable biomarkers and novel diagnostic/imaging techniques^1,72^. In addition to strengthening the relevance of the lymphatic system, these data highlight the need to promote translational and exhaustive research on it.

## Concluding remarks and future perspectives

There have been outstanding advances in understanding lymphatic endothelial metabolism. Accumulating evidence indicates that LECs acquire a unique metabolic signature during lymphangiogenesis, which is defined by: (1) active FAO, FA synthesis and KBO pathways to support cell proliferation and sprouting through the sustenance of the TCA cycle and nucleotide synthesis; (2) reliance on anaerobic glycolysis for energy production during proliferation and migration; (3) active OXPHOS for maintaining lymphatic identity; and (4) changes in amino acid metabolism. The metabolic mechanisms controlling lymphatic function are complex since almost all the metabolic pathways here reviewed are somehow interconnected with epigenetics and/or growth factor signaling pathways. Therefore, cellular metabolism should not be contemplated as an isolated set of pathways, but rather as a highly dynamic network. Still, many other metabolic pathways remain to be studied. For instance, it is yet unknown whether LECs, just like their blood counterparts and the rest of proliferative cells, are avid glutamine consumers and utilize this amino acid for biosynthetic, bioenergetic and redox modulatory purposes^70^. On the other hand, serine metabolism has been studied on BECs^67^, but no reports have been documented in LECs yet.

Findings in LEC metabolism are not just only meaningful for building a complete picture about the lymphatic vessel regulation under physiological conditions, but also for gaining a deeper understanding of this regulation in lymphatic-related diseases. Understanding the metabolic signature acquired by LECs during the establishment and progression of these pathologies will considerably increase the therapeutic opportunities and identify new potential targets that could synergize with conventional therapies, such as chemo-, radio- and immune-therapies. In the cancer context, while have been modestly addressed in BECs, further efforts are needed to decipher the metabolic crosstalk between LECs, cancer and other stromal cells and discover novel therapeutic targets for cancer immunomodulation. However, special attention should be paid when designing approaches targeting cellular metabolism; recent findings have revealed that permanent blockade of an entire central metabolic pathway (such as glycolysis) can induce systemic effects^148^. Instead, approaches based on vessel normalization should be considered to restore the metabolism of pathological LECs to that of normal LECs^118^.

Moreover, this field is benefiting from the emergence of cutting-edge technologies and multi-omics approaches, which allows for the massive comparative analysis within and between tissues. For instance, analyzing the transcriptomic profile of healthy LECs in different tissues^149,150^, or comparing LECs in pathological settings^151,152^, will help to identify promising therapeutic strategies. However, whether these molecular changes translate into metabolic peculiarities remains to be understood. In fact, insights into tissue-specific metabolic regulation of lymphatic vessels are completely underexplored. For example, the liver is the central organ for fatty acid metabolism. Therefore, liver ECs may be more specialized in the transport of fatty acids to support liver function. Understanding inter-organ signatures of LECs is of paramount importance to fully appreciate the regulation of vascular function for the design of more specific and effective strategies to treat vascular-related pathologies.
